# Co-Existence of Hereditary Pyrimidine 5’-Nucleotidase Deficiency and Heterozygous α-Thalassemia: A Case Presentation

**DOI:** 10.5505/tjh.2012.48642

**Published:** 2012-12-05

**Authors:** A. Agapidou, S. Theodoridou, K. Tegos, E. Mandala, E. Leukou, O. Karakasidou, B. Aletra, A. Sevastidou, M. Alemayehou, E. Voskaridou

**Affiliations:** 1 Hippokration Hospital of Thessaloniki, Hemoglobinopathy Prevention Unit, Thessaloniki, Greece; 2 Laikon Hospital of Athens, Thalassemia Center, Athens, Greece; 3 NIMITS, Military Hospital, Athens, Greece; 4 Aristotelion University of Thessaloniki, 4th Unit of Internal Medicine, Thessaloniki, Greece

**To the Editor,**

Pyrimidine 5’-nucleotidase (P5N) is an intra-erythrocytic isoenzyme that catalyzes the dephosphorylation of pyrimidine ribonucleotides, which are the product of DNA catabolism. A reduction in P5N reactivity leads to accumulation of pyrimidine products in erythrocytes, which increases its life span, resulting in hemolytic anemia and intense basophilic stippling [1]. 

P5N deficiency is inherited in an autosomal recessive fashion and is one of the most important causes of inherited non-spherocyte anemia, as well as G-6PD and pyruvate kinase deficiency. P5N deficiency is characterized by hemolytic anemia, splenomegaly, and intense basophilic stippling. There are several reports in the literature concerning reduced P5N reactivity in patients with intermediate and heterozygous β-thalassemia; however, the co-existence of P5N deficiency in a patient with α-thalassemia is a rare event [2,3,4]. 

We report the clinical and hematological findings in a 25-year-old female that was screened for hemoglobinopathies due to her pregnancy (6th week).Based on her medicalrecords, she was known to be a carrier of 5PN deficiency. The proposita’s hematological parameters were as follows: Hb: 10.7 g dL—1; Hct: 34%; RBC: 4.35 x 106; MCV: 77 fL; MCH: 24.7 pg; RDW: 16.2%; reticulocytes: 3.97%; ferritin: 30 (normal range=20-81ngr/Ml). Microscopic examination of a stained peripheral blood smear showed severe anisocytosis, microcytosis, and basophilic stippling. Biochemical analysis of hemoglobin using high-performance liquid chromatography (HPLC) showed normal values for hemoglobin A2 (HbA2: 2.4%) and hemoglobin F (HbF: 0.5%). No abnormal hemoglobins were detected. 

Due to an unclear hematological picture in the proposita’s husband, who was thought to be heterozygous for thalassemia, genetic analysis of the proposita’s DNA was performed. Molecular analysis showed that she was heterozygous for the mild mutation of α-thalassemia (-α3,7/ NI). In conclusion, mismatch between the hematological parameters of the presented pregnant patient and the usual hematological phenotype associated with α-thalassemia (-α 3,7/NI) were due to the lack of P5N (P5N: 1.88 U g–1 of Hb).The patient never required blood transfusion. The co-existence of hereditary P5N deficiency and heterozy-gous α-thalassemia is extremely interesting due to the fact that to the best of our knowledge this is the first report of such a case. 

**Conflict of Interest Statement**

The authors have no conflicts of interest, including specific financial interests, relationships, and/or affiliations, relevant to the subject matter or materials presented.
